# Estrogen and progesterone receptor status in breast cancer: a cross-sectional study of 450 women in Kerala, South India

**DOI:** 10.1186/1477-7819-12-120

**Published:** 2014-04-24

**Authors:** Gautham Rajan, Terence B Culas, PS Jayalakshmy

**Affiliations:** 1 Government Medical College, Thrissur, Kerala, India

**Keywords:** Breast cancer, estrogen receptor, progesterone receptor, premenopausal, postmenopausal, India

## Abstract

**Background:**

Hormone receptor status is an important prognostic and therapeutic tool in breast cancer. The objectives of our study were to create a database of breast cancer patients in Central Kerala between January 2010 and December 2012 and analyze the proportions of estrogen receptor and progesterone receptor positivity in premenopausal and postmenopausal women with breast cancer.

**Methods:**

Estrogen and progesterone receptor status were evaluated by immunohistochemistry. The chi-square test was used for statistical analysis.

**Results:**

The median age at diagnosis was 50 years while the mean age was 51.92 (SD = 11.78). 56.1% of premenopausal and 47.4% of postmenopausal patients were found to be ER positive, while PR positivity was 47.7% and 34.7% respectively in the premenopausal and postmenopausal age groups.

**Conclusions:**

The proportions of ER and PRnegative tumors were found to be lower than reported in earlier studies on Indian populations. Contrary to expectations, the proportions of ER and PRpositivity were found to be higher in the premenopausal age group.

## Background

Estrogen receptor (ER) status is an important predictive and prognostic factor in breast cancer, and determination of ER and progesterone receptor (PR) status of patients with breast carcinoma is now standard practice [1]. Though the incidence of breast cancer has been on a steady increase in Kerala [2], there have been very few studies of breast cancer in the state. This study was conducted in Government Medical College, Thrissur, a tertiary-care hospital in central Kerala. The objectives of our study were to create a database of patients in central Kerala who were detected to have breast cancer between January 2010 and December 2012 and to analyze the proportions of ER and PR positivity in premenopausal and postmenopausal women with breast carcinoma.

## Methods

The study was conducted between 1 January 2010 and 31 December 2012 utilizing data available at the Department of Pathology, Government Medical College, Thrissur. Female patients with histologically proven primary breast cancer who were treated at the Department of Surgery, Government Medical College, Thrissur were made part of the study. Patients whose hormone receptor status was not determined were excluded from the study. A total of 450 patients were studied.

All hormone receptor studies were done on post-mastectomy specimens, or tissue samples obtained by core-cut biopsy prior to anterior chemotherapy. In 2010 and 2011, ER status was determined using the BioGenex monoclonal mouse IgG1 (Clone 1D5) and PR status using BioGenex monoclonal mouse IgG1 (Clone 1A6). Antigen retrieval was done using the BioGenex EZ Retriever system. In 2012, DakoEnVision + System-HRP reagent was used for identification of antigens in paraffin-embedded tissues. Pressure cooking was used for antigen retrieval. Monoclonal mouse IgG1 (Clone 1D5) was the antibody used to study ER status. Monoclonal mouse IgG1 (Clone PgR 636) was used to determine PR status.

The premenopausal group consisted of patients who were 50 years or younger and the postmenopausal group of patients aged 51 years and older. The Early Breast Cancer Trialists’ Collaborative Group has used similar criteria to define menopause when menopausal status was not consistently defined across trials [3]. The median age at natural menopause has also been established to be 51 years [4,5]. The chi-square test was used for obtaining *P*-values and determining statistical significance. *P*-values lower than 0.05 were considered statistically significant.

## Results

Out of the 450 cases of breast cancer that were studied, 237 women were premenopausal and 213 were postmenopausal. The ages of the study subjects ranged from 26 to 90 years. The median age was 50 years. In the premenopausal age group ER was positive in 133 (56.1%) patients and PR was positive in 113 (47.7%). The number of premenopausal patients who were both ER and PRpositive was 102 (43.0%). In the postmenopausal age group of 213 patients, 101 (47.4%) were ERpositive and 74 (34.7%) were PRpositive: 72 patients (33.8%) were positive for both ER and PR. Detailed analysis by age is shown in Table 1.

**Table 1:** Age-wise analysis of study subjects

**Age group, years**	**Cases, number**	**ERpositive, number (%)**	**PRpositive, number (%)**	**Both ER and PRpositive, number (%)**	**Both ER and PRnegative, number (%)**
21 to 30	09	04 (44.4%)	05 (55.6%)	04 (44.4%)	04 (44.4%)
31 to 40	73	43 (58.9%)	37 (50.7%)	35 (47.9%)	28 (38.4%)
41 to 50	155	86 (55.4%)	71 (45.8%)	63 (40.6%)	61 (39.4%)
51 to 60	117	51 (43.6%)	40 (34.2%)	39 (33.3%)	65 (55.6%)
61 to 70	69	32 (46.4%)	23 (33.4%)	23 (33.3%)	37 (53.6%)
71 to 80	22	15 (68.2%)	10 (45.5%)	09 (40.9%)	06 (27.3%)
81 to 90	05	03 (60.0%)	01 (20.0%)	01 (20.0%)	02 (40.0%)

## Discussion

It is now well-established that ERpositive tumors are associated with better overall survival compared to ERnegative tumors [6]. Determination of the hormone receptor status has been routinely performed for breast cancer patients in our institution from the year 2008. Although the American Society of Clinical Oncology (ASCO) 2010 guidelines recommend that ER and PR assays be considered positive if at least 1% of tumor nuclei are positive in the sample being tested [7], earlier studies have used a 10% cutoff. Because the definition of hormone receptor positivity varies as mentioned, results obtained using the two criteria have been summarized in Table 2.

**Table 2:** Comparison of hormone receptor status based on American Society of Clinical Oncology (ASCO) 2010 and earlier criteria

**Hormone receptor status**	**10% cutoff**			**1% cutoff (ASCO 2010)**		
	**Pre-menopausal**	**Post-menopausal**	***P*-value**	**Pre-menopausal**	**Post-menopausal**	***P*-value**
**ERpositivity**	56.1%	47.4%	0.065	60.8%	54.5%	0.177
**PRpositivity**	47.4%	34.7%	0.005	58.7%	47.8%	0.022

ER, estrogen receptor; PR, progesterone, PR.

**Figure 1:**
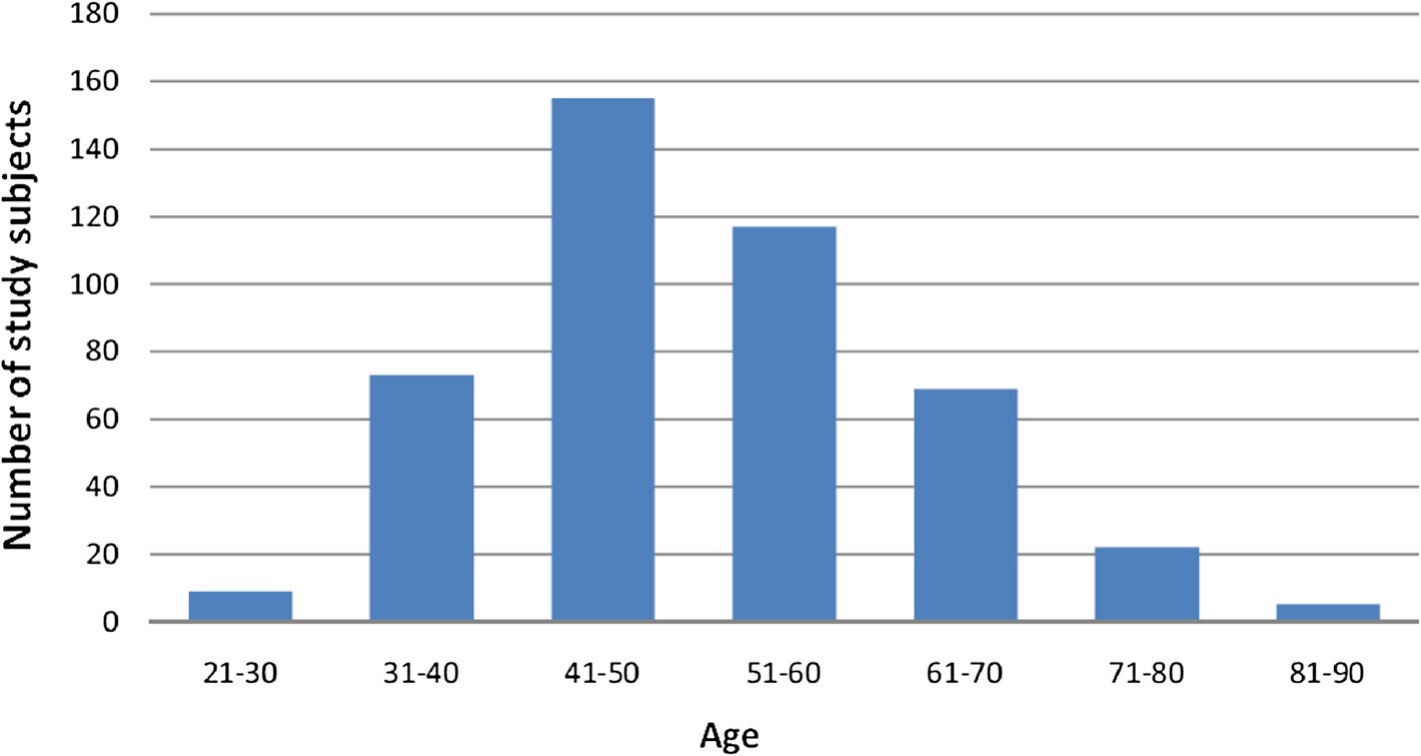
**Age distribution of study subjects.** The peak prevalence of breast cancer in central Kerala was found to be in the 41 to 50 years age group.

The peak prevalence of breast cancer in central Kerala was found to be in the 41 to 50 years age group (Figure 1). The median age at diagnosis was 50 years and the mean age was 51.92 (SD 11.78). In comparison, the median age at diagnosis for cancer of the breast in the US was 61 years [8]. Because the majority of patients in India present with a higher tumor-grade and a more advanced stage at diagnosis [9,10] when compared to those in the US [8], it is likely that the actual age of onset of breast carcinoma in the Indian patient is lower by well over a decade. This younger age at onset of breast cancer can in part be explained by racial differences [10].

Data from earlier studies conducted in India reveal a higher proportion of ERnegative breast cancers when compared to the US or Western Europe; the reasons for this are several - technical failures, younger age of patients, and a higher grade or advanced stage at presentation [8-13]. In our study, the overall ERpositivity was found to be 52.0%. The average ERpositivity for white women in the US is 77% [8]. Studies done in India and in Indian emigrants have found ERpositivity in Indian women to be 34.5% [14], 35.88% [12], 37.83% [15], 38.6% [11], 49.2% [13], 50.5% [9], and 55.1% [16]. ERpositivity in premenopausal and postmenopausal women was found to be 56.1% and 47.4% respectively. This difference was not found to be statistically significant (*P*-value 0.065). Only one earlier study done in India looks at the differences in ERstatus with respect to menopausal status; ERpositivity therein was found to be 23.1% in premenopausal women and 36.6% in postmenopausal women [14].

In our series 41.5% of patients were PRpositive while 55% of white women in the US are PRpositive [8]. Other studies from India put PRpositivity at 33.3% [11], 36.4% [14], and 42% [9]. The proportions of PRpositive tumors in the premenopausal and postmenopausal age groups were found to be 47.7% and 34.7% respectively (*P*-value 0.005). Kaul *et al*. in their study conducted in the Himalayan Region of Northern India found PRpositivity to be 23.1% and 39.0% respectively in premenopausal and postmenopausal women [14].

Ethnicity has been shown to be a factor in determining hormone receptor positivity. Studies in the US show that Asians have a lower ER positivity rate than non-Hispanic whites [17,18]. Our findings concur with these studies. There also appears to be geographical variations in ER positivity within India. The diet and lifestyle of our study population in Kerala, a state in the Southern tip of India with an expansive coastline, differs considerably from those of people in the North of the country. Seafood forms a prominent portion of the Kerala diet. Kerala stands first in the Human Development Index (HDI) among states in India, with an HDI of 0.790 (2011) against a national average of 0.467 (2011). Kerala also tops the country in every healthcare parameter, and awareness about breast cancer is also high among the people. These factors could explain the higher proportion of ER and PRpositive breast cancer in Kerala.

The immunohistochemistry technique used by our laboratory is standardized and is comparable with those used by other institutions in India and internationally [12-14,16]. Poor tissue handling or processing is unlikely to have altered the results obtained, as our institution has strict protocols and quality control measures.

## Conclusions

Contrary to what has been reported in earlier studies, we found the proportions of ER and PRpositivity to be higher in the premenopausal age group. Though India has a higher proportion of ERnegative breast cancer than the West, the situation is not as dismal as has been previously thought. Also, women in our series tend to be younger than their Western counterparts. Better tissue handling and processing will help decrease the number of false-negatives, and will ensure that hormonal manipulation is made available to a greater number of patients with breast cancer, thus improving the outcome of the disease.

The causes leading to the loss of the ER mechanism are currently being investigated, and if proven to be an epigenetic phenomenon, as has been postulated [19], it might in the near future be possible to convert ERnegative tumors to an ERpositive phenotype, thus bringing to an end the era of non-specific chemotherapy. Moreover, in a resource-limited setting like ours, a subset of premenopausal women who are ERpositive and have completed their families, but refuse adjuvant chemotherapy therapy can be offered the alternative option of surgical oophorectomy.

## Abbreviations

ER: estrogen receptor; PR: progesterone receptor; IgG: immunoglobulin; ASCO: American Society of Clinical Oncology; HDI: Human Development Index.

## Competing interests

The authors declare that we have no competing interests, financial or otherwise.

## Authors’ contributions

GR participated in the study design, collected and interpreted data, and prepared the manuscript. TBC conceived of and coordinated the study, participated in the study design, and revised the manuscript. PSJ carried out the immunohistochemical studies. All authors read and approved the final manuscript.
